# Gut Health Claims and Monetization Features on TikTok: Cross-Sectional Analysis

**DOI:** 10.2196/105391

**Published:** 2026-10-22

**Authors:** Kai Vu, Kyle Garcia-Rogers, Benjamin Li, Vinayak Swaroop, Owais Gilani, Alice M Tang, Michael Siegel

**Affiliations:** 1 Department of Public Health and Community Medicine School of Medicine Tufts University Boston, MA United States

**Keywords:** social media, TikTok, wellness, wellness influencers, medical influencers, health misinformation, gut health, gastroenterology

## Abstract

**Background:**

TikTok has emerged as a major source of health information, yet concerns persist regarding content accuracy and monetization features. Gut health content is particularly vulnerable to misinformation.

**Objective:**

This study aimed to examine the relationship between creator profession (“medical” vs “nonmedical”) and the quality of gut health claims and monetization features on TikTok.

**Methods:**

We conducted a cross-sectional study screening 412 unique TikTok creator accounts identified using the keywords “guthealth,” “gutcleansing,” and “digestion.” The final sample included 174 publicly accessible individual creators with at least 5000 followers and identifiable, relevant profession information. One video per creator was analyzed. Creator profession was categorized as medical or nonmedical, and health claim quality was coded as high, moderate, or poor. Monetization features (Showcase, Subscription, and external links) were assessed.

**Results:**

Nonmedical creators were more likely than medical creators to present poor- or moderate-quality health claims (adjusted prevalence ratio 2.33, 95% CI 1.50-3.62). Most creators (160/174, 92%) exhibited at least 1 monetization feature, and Showcase use was greater among nonmedical creators (adjusted prevalence ratio 1.57, 95% CI 1.02-2.42). Videos containing moderate- and poor-quality health claims received 3 times the expected likes as videos containing high-quality claims.

**Conclusions:**

Among the sampled creators, nonmedical creators were more likely to present poor- or moderate-quality gut health claims, and lower-quality claims were associated with higher like counts. These findings highlight a misalignment between information quality and like counts on social media.

## Introduction

### Background

Social media is increasingly becoming a primary source of health-related information among adolescents and young adults [1]. TikTok, a platform with nearly 1.9 billion monthly users as of early 2026, most of whom are aged between 18 and 34 years, has emerged as one of the most influential apps shaping health beliefs and behaviors [2]. Studies estimate that anywhere from 25% to more than 75% of adolescents and young adults view social media as a useful source of health information, often supplementing or replacing guidance from health professionals [3,4]. While social media platforms offer new opportunities for public health communication, growing evidence suggests that much of the health-related content circulated online is not evidence based and is frequently disseminated by noncredentialled individuals [5-8]. Content related to nutrition and digestive health has been identified as a particularly high-risk domain for misinformation, with viral videos commonly promoting detoxification practices, supplements, and restrictive diets that lack robust clinical evidence [9]. Exposure to misleading digestive health content may influence dietary behaviors, supplement use, and health care–seeking patterns, with potential downstream clinical consequences [10].

### Prior Work

Previous analyses of TikTok health content demonstrate that misleading or unverifiable claims made by creators often achieve equal or greater engagement than evidence-based information, presenting a major public health concern [11,12]. These patterns are not specific to creators within the public, as physicians and other medical creators have been shown to receive greater audience engagement from content containing poorer-quality health claims [12]. Many social media algorithms are designed to maximize engagement, which systematically privileges content that triggers attention—often moral or emotional and divisive content that overlaps with misinformation [13]. Popularity- and relevance-based platform algorithms have been identified as major drivers of online misinformation dissemination [14,15].

Despite growing recognition of this issue, important gaps remain in the literature. A limited number of papers have evaluated the quality of gastrointestinal health content on social media platforms [16-19]. Past studies have relied on small samples using a single search term, with limited evaluation of creator demographics, professional background, and various markers of professional credibility [5]. Furthermore, little research has directly examined how monetization strategies, such as commission-based affiliate marketing, external website promotion, or paid subscriptions, vary across creator professions (eg, “medical” vs “nonmedical” creators) on TikTok or how these strategies may influence the framing and credibility of health claims. Together, these limitations restrict understanding of how creator identity and monetization features interact to shape the quality and dissemination of health information on TikTok. Professional training may influence how creators evaluate and communicate health evidence, whereas monetization opportunities may incentivize product-oriented or attention-generating content. These factors may in turn shape the quality and framing of health claims presented to viewers. Because TikTok’s recommendation system incorporates user engagement in determining content visibility, highly engaging claims may achieve broad dissemination regardless of their evidentiary quality. Examining these factors together may therefore help clarify how creator characteristics and platform incentives contribute to the production and spread of gut health information.

### Study Objectives

To address these gaps, we conducted a cross-sectional study of TikTok videos related to gut health using multiple search queries and a standardized manual coding protocol. We examined whether creator professional background (medical vs nonmedical) was associated with the quality of health claims presented and with the presence of monetization features on creator profiles. In addition, we compared creator characteristics and platform features across professional groups to better understand differences in audience reach and presentation strategies. Finally, we assessed whether the quality of health claims in videos was associated with the number of likes received. We hypothesized that nonmedical creators would be more likely than medical creators to present lower-quality health claims and use monetization features and that lower-quality health claims would attract greater audience engagement.

## Methods

### Data Collection and Study Eligibility

Data collection followed a standardized process. First, a new TikTok account was created with no prior search or watch history to minimize personalization of search results and reduce potential algorithmic bias from prior user activity. Creator usernames were then identified through platform search queries using the terms “guthealth,” “gutcleansing,” and “digestion.” Searches were conducted on December 12, 2025 (“guthealth” and “gutcleansing”), and January 20, 2026 (“digestion”). The third search query was added to capture a broader range of gastrointestinal health content, including content produced by medical creators. For this study, gut health encompassed general digestive wellness, gastrointestinal symptoms and diagnosed conditions, the gut microbiome, and diets or supplements presented as affecting digestive or gut health. Each search was conducted once, in the order listed, using the default “Relevance” sorting. Reviewers scrolled through all available results until no additional videos were returned by the platform and recorded the associated creator usernames in separate lookup sheets by search term. No investigator-defined numerical stopping threshold was applied. Duplicate creators were identified within and across searches, and only the first video encountered from each creator was retained. This video was designated the “indexed video” for subsequent coding; additional results from the same creator were excluded regardless of whether they represented the same or different videos. Each unique username was assigned a numeric study ID, and the username-ID linkage file was stored separately from the analytic dataset.

The lead author developed the coding framework and trained the other 3 reviewers in its application. All 4 reviewers independently coded a randomly selected 10% subsample, and interrater agreement was assessed using Fleiss κ before proceeding with the remaining data collection. The lead author’s ratings for this subsample were retained in the analytic dataset. The remaining accounts were distributed approximately equally among the 4 reviewers, with each account and indexed video assessed by 1 reviewer. Reviewers accessed videos and corresponding creator profiles through the TikTok mobile app and recorded observations in a dataset containing only study IDs and study variables. Data collection began on December 20, 2025, and was completed on February 16, 2026.

Inclusion criteria were (1) publicly accessible TikTok creator accounts identified through predefined search queries, (2) accounts representing individual content creators, and (3) accounts with at least 5000 followers. Accounts for which the creator or indexed video could not be located, as well as those with unavailable or unrelated profession information, were subsequently excluded.

### Measures

Data were collected on a combination of video- and account-level variables for each creator. For each video identified in our search, the number of likes, date posted, and whether subtitles were present were recorded. Video dates were further categorized by calendar year and quarter (ie, January-March and April-June). The following creator account-level characteristics associated with each respective video were recorded: observed gender (man, woman, or indeterminate or other); observed age (<40 years or ≥40 years); verification status (whether the user had an official TikTok verification badge); follower count; total like count across all account videos; and whether the full name and/or “Doctor” (or “Dr.”) prefix was used in the account name, username, or biography.

Creator profession was coded based on the credentials, title, and role presented on the creator’s TikTok profile (eg, username; biography; and linked content, such as Linktrees) and other supplemental accessible public-facing materials that were identified as associated with the creator based on their TikTok profile when necessary (eg, LinkedIn with the same profile picture, affiliated social media accounts with the same profile username, and National Provider Identifier Registry with the same location in profile biography). Profession categories included physicians, nurse practitioners, scientists (including researchers and professors), dentists, other professionally prepared clinicians, chiropractors, alternative and functional medicine specialists, and health coaches and influencers. Physicians were defined as both US (MD and DO) and international (MD, MBBS, MBChB, etc) medical graduates. Physician-presenting profiles that could not be verified via the National Provider Identifier Registry or other public-facing materials were categorized as “unverified ‘doctors’.” “Other professionally prepared clinicians” were defined as clinical professions requiring professional or graduate degrees and included physician assistants, occupational therapists, physical therapists, podiatrists, optometrists, perfusionists, licensed therapists, mental health counselors, social workers, audiologists, and registered dietitians. “Alternative or functional medicine specialists” included naturopathic doctors, naturopaths, functional medicine specialists, acupuncturists, and related alternative providers. “Health coaches and influencers” included wellness influencers, coaches, personal trainers, and nutritionists who were not registered dietitians. Professions were recoded dichotomously for analysis as medical creators (physicians, nurse practitioners, dentists, scientists, and other professionally prepared clinicians) or nonmedical creators (chiropractors, alternative and functional medicine specialists, and health coaches and influencers). Licensure or certification was determined by whether the creator presented evidence of professional licensure, board certification, or other professional credentials on their profile.

Each video was reviewed to assess the quality of the health claim present and was coded in 1 of 4 categories: poor, moderate, high, or no claim present. Poor-quality claims were characterized by harmful or potentially misleading information and were defined as unsubstantiated or overstated medical claims lacking supporting evidence, contradicting accepted guidelines, relying on anecdotes or pseudoscience, using fearmongering, or lacking biological plausibility. Moderate-quality claims were defined as partially substantiated or plausible claims that were exaggerated or overgeneralized based on weak evidence or lacking appropriate context regarding risks or limitations. High-quality claims were defined as well-substantiated and evidence-based claims presented responsibly with acknowledgment of limitations when applicable and without exaggeration. Health claim quality was further recoded into a dichotomous variable (high vs poor or moderate quality) for some analyses. When reviewers were uncertain about a claim’s evidentiary support, they entered the creator’s exact wording into OpenEvidence (Xyla) and reviewed both the generated summary and cited literature. Reviewers then applied the study’s coding criteria to assign the claim-quality category [20]. Videos with content unrelated to health or that had no health-relevant takeaways were categorized as no claim present.

Creator monetization features were assessed using 3 separate dichotomous measures (present or not present) directly from the account profile page: “Showcase,” “Subscription,” and the presence of external links with potential for monetization. Showcase was coded as present if the creator had a TikTok Showcase available on their profile, which allows creators to receive commission from linked product purchases (either directly from videos or from the account profile) through TikTok Shop. Subscription was coded as present if the creator offered a subscription on their profile, an optional paid feature that viewers can purchase to access exclusive videos, tiered badges, and other creator-specific perks. An external link was coded as present if the creator included links to external websites, including but not limited to Linktree, Shopify, merchandise, personal websites, courses, appointments, or blog posts. These variables were also combined into a dichotomous composite variable indicating the presence of at least 1 assessed monetization feature.

### Statistical Analysis

All analyses were performed in R (version 4.5.2; R Project for Statistical Computing) [21]. Creator account and video characteristics were summarized descriptively. Bivariate analyses compared select characteristics by creator profession (ie, nonmedical vs medical) using the Wilcoxon rank-sum and chi-square tests for continuous and categorical variables, respectively. Select characteristics, including doctor prefix, verification status, full name use, and licensure or certification, were included as indicators of professional authority and credentials.

Our primary objective was to assess the relationship between creator profession and health claim quality. Health claim quality was analyzed dichotomously (poor or moderate quality vs high quality). We generated crude and adjusted prevalence ratios (PRs), comparing the prevalence of poor- and moderate-quality vs high-quality health claims by creator profession. Estimates were calculated using Poisson regression with robust standard errors. The adjusted model controlled for age, gender, year, quarter, and search query. We adjusted for search query because different search terms could retrieve creators with different professional backgrounds and claim characteristics. Posting year and quarter were included to account for temporal differences in the videos represented in the sample. Visually assessed age and gender were included as exploratory creator characteristics that might be associated with both profession and claim quality; their roles as confounders were uncertain. Given the low interrater agreement for observed age (κ=0.15), we repeated the analysis without that variable.

We also assessed the association between creator profession and monetization features. Crude and adjusted models were fit using 4 measures of monetization features: Showcase, Subscription, external link, and any of the aforementioned features. Our adjusted models again controlled for age, gender, year, quarter, and search query.

Finally, we examined the association between health claim quality and the number of video likes received. Crude and adjusted negative binomial regression models were fitted to estimate incidence rate ratios (IRRs) for video likes across claim quality categories. In this analysis, health claim quality was modeled as a 3-level categorical variable, with high-quality claims as the reference category. The adjusted model controlled for age, gender, year, quarter, search query, number of account followers, presence of subtitles in the video, and Showcase presence. The latter 3 variables were included due to being visible at the video level and thus potentially confounding this association. Finally, we conducted an interaction analysis to assess whether the association between health claim quality and video likes differed between medical and nonmedical creators. We selected negative binomial regression because video likes are a count outcome, and substantial variation in engagement makes overdispersion plausible. Unlike Poisson regression, negative binomial regression allows the conditional variance to exceed the conditional mean.

Interrater reliability was assessed using Fleiss κ among a 10% subsample coded individually by all 4 reviewers for select categorical variables requiring subjective interpretation. To account for potential misclassification bias, 2 post hoc sensitivity analyses were performed: one excluding unverified “doctors” from the nonmedical creator subgroup and another categorizing unverified “doctors” as medical creators, with primary and secondary objective analyses repeated under both approaches. In a third sensitivity analysis, we fitted separate adjusted modified Poisson models comparing moderate- vs high-quality claims and poor- vs high-quality claims, restricting each analysis to the corresponding 2 categories. In a final post hoc sensitivity analysis, we replaced posting year and quarter with log-transformed video age (in days), calculating video age as the number of days between posting and the query-specific search date, to account more directly for differences in the time available to accumulate likes.

### Ethical Considerations

The study protocol was reviewed by the Tufts Health Sciences Institutional Review Board and determined not to constitute human subjects research (STUDY00006621).

## Results

### Sample Identification and Study Population

The 3 searches yielded 509 creator account entries before deduplication, representing 412 unique creator accounts screened for eligibility (Figure 1). Of these, 238 accounts were excluded because the creator or indexed video could not be identified (n=41, 17%), the account was not an individual creator account (n=64, 27%), profession was unavailable or unrelated (n=100, 42%), or the creator had fewer than 5000 followers (n=33, 14%). The final analytic sample included 174 creators.

**Figure 1:**
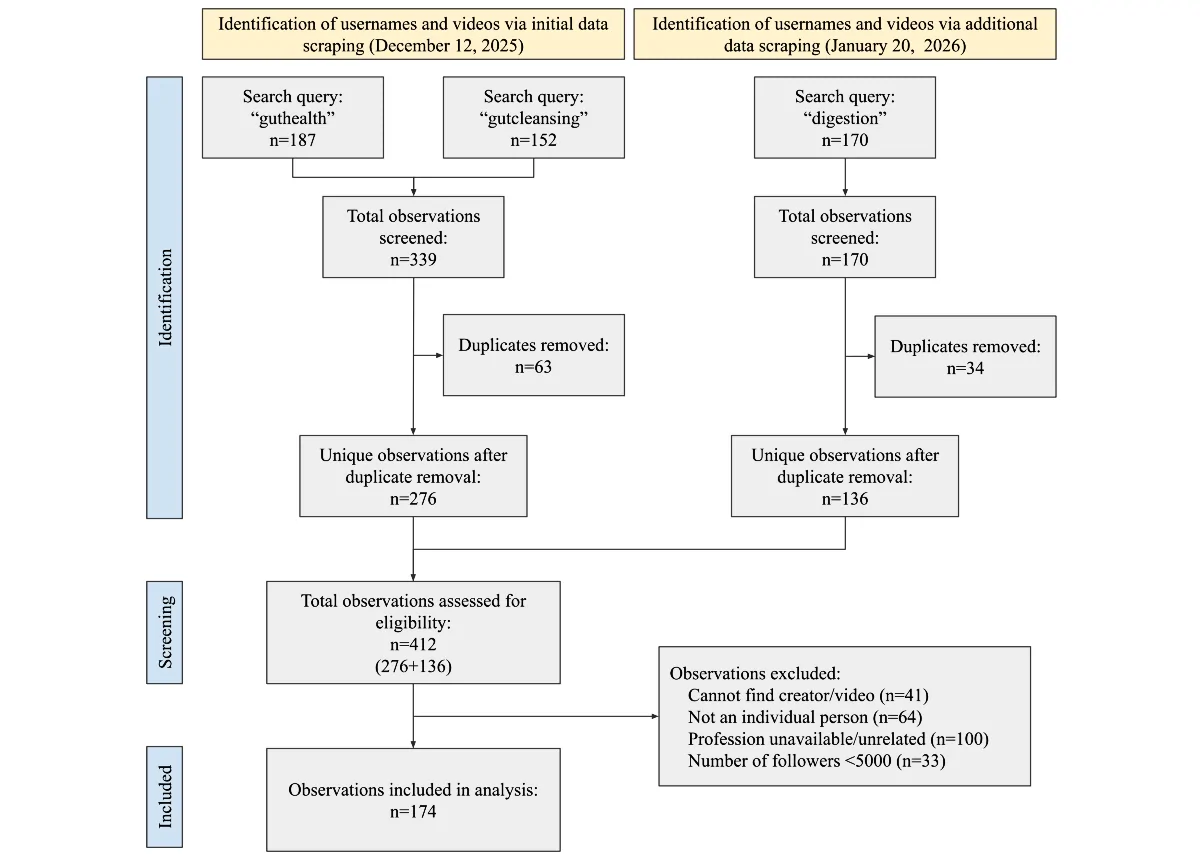
Data collection timeline and study selection flowchart.

### Creator Account and Video Characteristics

Of 174 TikTok creators in the final sample, 29 (17%) were medical creators and 145 (83%) were nonmedical creators (Table 1). Among 29 medical creators, the most common professions were physicians (n=15, 52%) and other professionally prepared clinicians (n=8, 28%), whereas of 145 nonmedical creators, the overwhelming majority were health coaches and influencers (n=130, 90%). A detailed list of credentials by profession is provided in Table S1 in Multimedia Appendix 1.

**Table 1:** Characteristics by creator profession (N=174).

Characteristics			Total		Medical creators (n=29)		Nonmedical creators (n=145)		*P* value^a^			κ^b^	
**Profession, n (%)**										—^c^			0.83
	Physician	15 (8.6)		15 (52)		N/A^d^							
	Nurse practitioner	2 (1.1)		2 (6.9)		N/A							
	Scientist, researcher, or professor	2 (1.1)		2 (6.9)		N/A							
	Other professionally prepared clinician	8 (4.6)		8 (28)		N/A							
	Chiropractor	1 (0.6)		N/A		1 (0.7)							
	Alternative or functional medicine specialist	8 (4.6)		N/A		8 (5.5)							
	Health coach or influencer	130 (75)		N/A		130 (90)							
	Unverified “doctor”	6 (3.4)		N/A		6 (4.1)							
	Other^e^	2 (1.1)		2 (6.9)		N/A							
Official verification status, n (%)			14 (8)		7 (24)		7 (4.8)		.002			0.90	
**Observed gender, n (%)**										>.90			1.00
	Man	54 (31)		9 (31)		45 (31)							
	Woman	120 (69)		20 (69)		100 (69)							
Age ≥40 years, n (%)			49 (28)		13 (45)		36 (25)		.05			0.15	
Total account followers, median (IQR)			90,700 (28,400-304,200)		163,600 (55,700-952,000)		73,300 (27,800-275,500)		.01			—^f^	
Total account likes, median (IQR)			1,500,000 (426,200-6,700,000)		2,700,000 (667,900-16,100,000)		1,400,000 (425,000-5,100,000)		.12			—	
**Video characteristics** ^g^													
	Number of likes, median (IQR)	18,200 (3906-55,100)		8574 (2822-29,600)		22,400 (4613-76,200)		.08			—		
	Subtitles present, n (%)	90 (52)		16 (55)		74 (51)		.80			—		
**Monetization features** ^g^ **, n (%)**													
	Any	160 (92)		25 (86)		135 (93)		.40			—		
	Showcase	103 (59)		12 (41)		91 (63)		.05			1.00		
	Subscription	59 (34)		9 (31)		50 (34)		.90			1.00		
	External link	122 (70.1)		21 (72)		101 (70)		>.90			0.62		
Licensed or certified, n (%)			29 (17)		19 (66)		10 (6.9)		<.001			0.81	
Full name in profile, n (%)			57 (33)		12 (41)		45 (31)		.40			0.87	
Doctor prefix in profile, n (%)			25 (15)		15 (51.7)		10 (6.9)		<.001			1.00	
**Search query, n (%)**										.60			—
	“Digestion”	29 (17)		5 (17)		24 (17)							
	“Gutcleansing”	53 (30)		11 (38)		42 (29)							
	“Guthealth”	92 (53)		13 (45)		79 (54)							
**Year of video posting, n (%)**										.40			—
	2020	1 (0.6)		0 (0)		1 (0.7)							
	2021	3 (1.7)		1 (3.4)		2 (1.4)							
	2022	6 (3.4)		2 (6.9)		4 (2.8)							
	2023	20 (11)		3 (10)		17 (12)							
	2024	38 (22)		10 (34)		28 (19)							
	2025	104 (60)		13 (45)		91 (63)							
	2026	2 (1.1)		0 (0)		2 (1.4)							
**Quarter of video posting, n (%)**										.20			—
	January-March	36 (21)		8 (28)		28 (19)							
	April-June	46 (26)		6 (21)		40 (28)							
	July-September	54 (31)		12 (41)		42 (29)							
	October-December	38 (22)		3 (10)		35 (24)							
**Health claim quality, n (%)**										<.001			0.41
	None	10 (5.7)		2 (6.9)		8 (5.5)							
	Poor	92 (53)		5 (17)		87 (60)							
	Moderate	51 (29)		6 (21)		45 (31)							
	High	21 (12)		16 (55)		5 (3.4)							

^a^Calculated using the χ^2^ test for categorical variables and the Wilcoxon rank-sum test for continuous variables.

^b^Calculated using Fleiss κ.

^c^Not calculated because this variable was used for stratification.

^d^N/A: not applicable.

^e^Other includes “high school science teacher” (n=1) and “graduate student” (n=1).

^f^ —: not calculated because this variable did not require subjective coding.

^g^Subcategory percentages are nongranular and do not sum to 100%.

Medical creators were more likely than nonmedical creators to have verification badges (7/29, 24% vs 7/145, 4.8%; *P*=.002), list licensure or certification (19/29, 66% vs 10/145, 6.9%; *P*<.001), and use a doctor prefix in their profiles (15/29, 51.7% vs 10/145, 6.9%; *P*<.001). Medical creators also had a higher median follower count than nonmedical creators (163,600 [IQR 55,700-952,000] vs 73,300 [IQR 27,800-275,500]; *P*=.01). Median total account likes were higher among medical creators (2,700,000 [IQR 667,900-16,100,000] vs 1,400,000 [IQR 425,000-5,100,000]), but the difference was not statistically significant (*P*=.12). Monetization features were highly prevalent and did not differ statistically significantly between groups (25/29, 86% vs 135/145, 93%; *P*=.40).

Indexed videos differed statistically significantly in health claim quality by creator type (*P*<.001): high-quality claims were most common among medical creators (16/29, 55%), whereas poor-quality claims were most common among nonmedical creators (87/145, 60%). Interrater reliability varied across subjectively coded variables. Agreement was slight for observed age (κ=0.15), moderate for health claim quality (κ=0.41), and substantial to almost perfect for the remaining assessed variables (κ range 0.62-1.00).

### Creator Profession and Health Claim Quality

Compared with medical creators, nonmedical creators were more than twice as likely to produce poor- or moderate-quality health claims in their videos (adjusted PR 2.33, 95% CI 1.50-3.62; Table 2). Of 137 nonmedical creators, only 5 (3.6%) produced high-quality health claims. Excluding observed age produced a similar association between nonmedical creator status and poor- or moderate-quality claims (adjusted PR 2.35, 95% CI 1.51-3.65), compared with the primary adjusted estimate of 2.33 (95% CI 1.50-3.62; Table S2 in Multimedia Appendix 1).

**Table 2:** Association between creator profession and health claim quality (N=164)^a^.

Health claim quality^b^	Profession, n (%)			PR^c^ (95% CI)	
	Medical creators (n=27)	Nonmedical creators (n=137)	Crude		Adjusted^d^
High	16 (59.3)	5 (3.6)	Reference		Reference
Poor or moderate	11 (40.7)	132 (96.4)	*2.36 (1.5-3.73)* ^e^		*2.33 (1.50-3.62)*

^a^PRs compare the prevalence of poor- or moderate-quality claims among nonmedical vs medical creators.

^b^Ten excluded due to not having health claims present in the indexed video.

^c^PR: prevalence ratio.

^d^Adjusted for age, gender, year, quarter, and search query.

^e^Italics indicate statistical significance.

### Creator Profession and Monetization Features

Compared with medical creators, nonmedical creators had a higher prevalence of the Showcase feature on their profiles (adjusted PR 1.57, 95% CI 1.02-2.42; Table 3). The adjusted association was statistically significant, whereas the crude association was not. No associations were identified between creator profession and the presence of subscriptions or external links.

**Table 3:** Crude and adjusted associations between creator profession and monetization features (N=174).

Monetization feature	Profession, n (%)			PR^a^ (95% CI)	
	Medical creators (n=29)	Nonmedical creators (n=145)	Crude		Adjusted^b^
Any	25 (86.2)	135 (93.1)	1.08 (0.93-1.26)		1.09 (0.94-1.27)
Showcase	12 (41.4)	91 (62.8)	1.52 (0.97-2.38)		*1.57 (1.02-2.42)* ^c^
Subscription	9 (31)	50 (34.5)	1.11 (0.62-2.00)		1.14 (0.66-1.98)
External link	21 (72.4)	101 (69.7)	0.97 (0.76-1.24)		0.98 (0.76-1.28)

^a^PR: prevalence ratio.

^b^Adjusted for age, gender, year, quarter, and search query.

^c^Italics indicate statistical significance.

### Health Claim Quality and Video Engagement

Compared with high-quality claims, videos containing moderate- or poor-quality claims received more than 3 times the expected likes (Table 4). Specifically, moderate-quality claims were associated with 3.16 times as many expected likes (adjusted IRR 3.16, 95% CI 1.30-7.26), and poor-quality claims were associated with 3.37 times as many expected likes (adjusted IRR 3.37, 95% CI 1.41-7.51). No statistically significant interaction was observed between creator profession and health claim quality in relation to video likes.

**Table 4:** Association between health claim quality and video likes (N=164).

Predictor		Ratio of expected likes, IRR^a^ (95% CI)		
		Crude	Adjusted^b^	Interaction^c^
**Claim quality^d^**				
	High (n=21)	Reference	Reference	—^e^
	Moderate (n=51)	*3.04 (1.27-6.63)* ^f^	*3.16 (1.30-7.26)*	—
	Poor (n=92)	*3.8 (1.66-7.72)*	*3.37 (1.41-7.51)*	—
**Interaction with profession**				
	Moderate×nonmedical professional	—	—	0.36 (0.06-2.21)
	Poor×nonmedical professional	—	—	1.18 (0.18-7.80)

^a^IRR: incidence rate ratio.

^b^Adjusted for age, gender, year, quarter, search query, number of account followers, video subtitles, and whether the account had a Showcase.

^c^Interaction terms represent the ratios of the claim quality IRRs among nonmedical vs medical creators.

^d^Ten excluded due to not having health claims present in the indexed video.

^e^Not applicable.

^f^Italics indicate statistical significance.

### Sensitivity Analysis

Sensitivity analyses excluding 6 unverified “doctors” yielded findings similar to the primary analyses; however, the crude association between creator profession and Showcase use became statistically significant (crude PR 1.58, 95% CI 1.01-2.48; Tables S3 and S4 in Multimedia Appendix 1). When unverified “doctors” were recategorized as medical creators, the magnitude of the association between creator profession and health claim quality decreased slightly (adjusted PR 1.88, 95% CI 1.37-2.58; Table S5 in Multimedia Appendix 1). In addition, both the crude and adjusted associations between profession and any monetization feature present became statistically significant, with nonmedical creators being more likely to have any form of analyzed monetization feature present (adjusted PR 1.24, 95% CI 1.05-1.48; Table S6 in Multimedia Appendix 1). The pairwise sensitivity analyses comparing moderate- vs high-quality claims and poor- vs high-quality claims yielded associations consistent with the primary analysis (Table S7 in Multimedia Appendix 1). Finally, as an alternative measure to account more directly for differences in the time since a video had been posted (and available to accumulate likes) before being recorded, we replaced posting year and quarter with log-transformed video age (in days), calculating video age relative to the query-specific search date. Associations remained statistically significant: compared with high-quality claims, moderate- and poor-quality claims were associated with expected like counts 3.59 times as high (95% CI 1.63-7.93) and 4.20 times as high (95% CI 1.98-8.91), respectively (Table S8 in Multimedia Appendix 1).

## Discussion

### Principal Findings

Our study provides evidence that, within the gut health and wellness space on TikTok, nonmedical creators are more likely than medical creators to present poorer-quality health claims and have a Showcase on their profiles. This cross-sectional analysis is, to our knowledge, the first to directly analyze differences in health claim quality and monetization features by creator profession in social media content. In addition, videos containing poorer-quality gut health claims received more than 3 times the expected number of likes compared with high-quality claims.

These findings are concerning, given the scale of the TikTok user base and the potential for large-scale dissemination of misinformation, particularly for viewership and financial gain. The association between lower claim quality and higher like counts raises questions about how audiences encounter and respond to gut health information. However, our data cannot distinguish greater exposure from a greater propensity to like a video after viewing it, nor can they establish the role of creator financial incentives or recommendation algorithms.

### Comparison With Prior Work

Interestingly, we did not observe a statistically significant effect of profession on the association between health claim quality and video likes. However, this may reflect limited statistical power, as only 5 and 6 medical creators had poor- and moderate-quality health claims, respectively. Previous studies have demonstrated this negative association between health claim quality and audience engagement among both medical and nonmedical creators [11,12]. Whether the strength of this relationship differs between groups remains unclear and warrants further investigation.

Although most creators in our sample exhibited at least 1 monetization feature, we observed only 1 statistically significant association between creator profession and monetization: nonmedical creators were more likely than medical creators to list a Showcase in their profile (63% vs 41%). External links were included as a proxy for potential monetization; however, not all links directly generate revenue. For example, a medical creator linking to the Centers for Disease Control and Prevention may be classified as having an external link despite having no financial intent. This may have resulted in misclassification and attenuated associations involving this measure. Similarly, TikTok Subscriptions were initially introduced in 2022 as an invite-only feature and only became widely available near the end of 2024 [22]. As a relatively new and evolving feature, their uptake remains limited, and associations with creator profession may be unstable and require reassessment over time. In contrast, Showcases, which allow creators to link products from TikTok Shop and earn commissions, represent a more direct measure of monetization. Given the growing integration of TikTok Shop into the platform and its role in facilitating product-based sales, Showcase may be particularly relevant in gut health content, where supplements and wellness products are commonly marketed. While our measure captured the presence of a Showcase at the profile level rather than whether the identified video included a product link, this likely reflects a creator’s overall engagement in product-based monetization. Taken together, these considerations suggest that Showcases may represent a more robust indicator of monetization features, supporting the observed association with creator profession.

### Limitations

This study has several limitations. Health claim quality was assessed using a structured coding framework developed for this study rather than a previously validated tool. Few validated instruments have been used to evaluate short-form health content; although tools such as DISCERN have been used in other studies analyzing short-form content, these tools were designed and validated specifically for written health information [23]. To our knowledge, no validated scale currently exists specifically for evaluating health claims in short-form videos. Interrater agreement for health claim quality was moderate (κ=0.41), indicating potential uncertainty in claim classification. The original DISCERN validation study reported weighted κ values of 0.53 among expert panelists and 0.40 among professional information providers [24], although differences in agreement statistics and assessment settings limit direct comparison. Estimated OpenEvidence use varied between 30% and 50% among reviewers, and the evidence used to inform classification was not independently verified by a qualified health professional. These limitations may have contributed to the misclassification of health claim quality.

Reviewer bias may have influenced the classification of health claims, with reviewers potentially appraising content from medical creators more favorably. Therefore, our coding approach emphasized biological plausibility, support from peer-reviewed literature, and potential for harm rather than strict concordance with clinical guidelines alone. Health claim quality was assessed before account-level characteristics were recorded; however, reviewers were not blinded to professional cues visible in videos or profiles. Similarly, age and gender were visually assessed, and agreement for age was low; residual confounding or measurement error in these variables remains possible. Misclassification bias is also a concern, given our sampling strategy. Duplicate creators appearing within and across search queries were removed so that each creator contributed only 1 video to the analytic sample. This approach prevented highly visible creators from being overrepresented, but only the first indexed video from each creator was analyzed. As a result, the selected video may not reflect the overall quality or tone of the creator’s broader content. The selected video may also not accurately represent the average level of audience engagement across multiple videos from a single creator, given the relevance-based nature of TikTok’s search algorithm.

In addition, the sample was limited to publicly accessible individual creator accounts with at least 5000 followers that were identified through a limited number of predefined search terms. Different gut health–related search queries, smaller creators, private accounts, or organizational accounts may have produced different distributions of creator professions, health claim quality, monetization features, and engagement. Because the “digestion” search occurred more than a month after the other searches, changes in platform trends or search behavior may have affected which creators and videos were retrieved. Search term and search date could not be disentangled in this study. Excluding 100 accounts because profession information was unavailable or unrelated may also have introduced selection bias toward creators who explicitly identified their professional credentials or influencer status. Because professional background was the primary exposure, this selection may have influenced the observed distribution of creator groups and their associations with claim quality and monetization indicators. Furthermore, unverified “doctors”—creators using “Doctor” or “Dr.” prefixes without easily identifiable evidence of medical training—were categorized as nonmedical, potentially introducing misclassification bias. To address this concern, we conducted sensitivity analyses excluding these creators, which did not reveal appreciable differences in our results. However, residual misclassification remained possible when professional identity could not be fully confirmed from public information. In addition, measures of monetization features were also limited to visible, profile-level features and may not reflect undisclosed sponsorships or other revenue streams outside TikTok, thus potentially underestimating creators’ monetization features. TikTok only began stricter enforcement of its brand disclosure policy in late 2025. Sponsored posts, undisclosed partnerships, affiliate links embedded in videos, and external commercial arrangements may not have been captured [25].

Residual bias remains with other readily measurable variables; video duration and posting frequency are feasibly measured confounders that could strengthen future analyses but were not collected in this study. Other factors, such as production quality, sentiment, emotional framing, topic complexity, and audience engagement metrics (eg, posting frequency and hashtag use), may confound the associations in our analyses but would require additional operational definitions and systematic coding that are beyond the scope of this study. Accordingly, our findings should be interpreted as associations rather than causal effects.

TikTok’s search and recommendation systems are proprietary, dynamic, and not fully reproducible [26]. We attempted to mitigate the “black box” nature of the algorithm by minimizing personalization; a new TikTok account was created with no prior search or watch history. However, search results may still have been influenced by geographic factors, device-level signals, timing, or other algorithmic features. Because TikTok accounts require an email address for registration, the use of a preexisting email account with an institution-affiliated domain could have potentially introduced residual personalization or algorithmic bias. Accordingly, the analyzed content may not be representative across geographic, temporal, or algorithmic contexts. Content generalizability is also limited because gut health and wellness represent a particularly monetizable niche in which supplements, cleansing, detoxification, and microbiome-related claims are common. The effect size of our associations may not generalize to other lucrative areas of digital health misinformation, such as dermatology, psychiatry, or reproductive health; further research is needed to evaluate the quality of online health information in these areas. Similarly, results may not generalize to other platforms, including Instagram, YouTube, or long-form websites, where content length, user engagement, and monetization potential differ substantially. Future studies with larger sample sizes and/or more search queries would also allow for more granular analysis between individual professions within each subgroup category.

The observed associations have practical implications for evaluating gut health information on TikTok. Nonmedical creators had 2.33 times the adjusted prevalence of poor- or moderate-quality claims compared with medical creators, although professional background alone did not guarantee claim quality. Videos containing moderate- and poor-quality claims had approximately 3 times the expected number of likes compared with high-quality claims, indicating that popularity should not be treated as a marker of accuracy. These findings support prioritizing highly liked gut health content for claim-level quality assessment across professional groups. Potential platform approaches, such as evidence labels and clearer presentation of professional credentials, have been implemented elsewhere and warrant further evaluation for their feasibility as tools to combat health misinformation on TikTok [27]. Future research should also develop and validate instruments for assessing short-form health content and evaluate whether automated natural language processing or multimodal methods can reliably complement manual coding.

### Conclusions

Among the sampled TikTok creators, nonmedical creators were more likely than medical creators to present poor- or moderate-quality gut health claims. Lower-quality claims were associated with higher expected like counts, indicating that audience engagement did not align with claim quality. Profile-level monetization indicators were common across professional groups, with Showcase use more prevalent among nonmedical creators. These findings support further quality assessment of highly liked gut health content and evaluation of interventions to help users assess the evidence underlying health claims.

## Appendix

Multimedia Appendix 1 Additional methodological information and sensitivity analyses evaluating the robustness of associations between creator profession, health claim quality, monetization features, and video likes.

## Abbreviations

IRR: incidence rate ratio
PR: prevalence ratio

## Data Availability

The data that support the findings of this study are openly available in Harvard Dataverse [28].
