# Supplementary material for: Gut Health Claims and Monetization Features on TikTok: Cross-Sectional Analysis
Source: Online J Public Health Inform. 2026 Oct 22;18:e105391. doi: 10.2196/105391 (PMC13740816; doi:10.2196/105391)
Supplement: Supplementary file 1 [file ojphi_v18i1e105391_app1.docx]

**Supplementary Table 1.** Credentials by creator profession (N = 174)

| Profession^†^ | Total,  N = 174 | Medical Creators,  N = 29 | Non-Medical Creators,  N = 145 |
| --- | --- | --- | --- |
| Physician | 5 (2.9%) | 5 (17%) | — |
| Physician (DO) | 2 (1.1%) | 2 (6.9%) | — |
| Physician (MD) | 7 (4.0%) | 7 (24%) | — |
| Physician (MD, FACS) | 1 (0.6%) | 1 (3.4%) | — |
| Scientist/Researcher/Professor | 1 (0.6%) | 1 (3.4%) | — |
| Scientist/Researcher/Professor (PhD, BSc, BEd) | 1 (0.6%) | 1 (3.4%) | — |
| Nurse Practitioner | 1 (0.6%) | 1 (3.4%) | — |
| Nurse Practitioner (FNP) | 1 (0.6%) | 1 (3.4%) | — |
| Other Professionally-Prepared Clinician | 6 (3.4%) | 6 (21%) | — |
| Other Professionally-Prepared Clinician (PT, BS) | 1 (0.6%) | 1 (3.4%) | — |
| Other Professionally-Prepared Clinician (RDN) | 1 (0.6%) | 1 (3.4%) | — |
| Other^‡^ | 2 (1.1%) | 2 (6.9%) | — |
| Health Coach/Influencer | 126 (72%) | — | 126 (87%) |
| Health Coach/Influencer (BS) | 1 (0.6%) | — | 1 (0.7%) |
| Health Coach/Influencer (LMT) | 1 (0.6%) | — | 1 (0.7%) |
| Health Coach/Influencer (MOMsSc) | 1 (0.6%) | — | 1 (0.7%) |
| Health Coach/Influencer (TTS) | 1 (0.6%) | — | 1 (0.7%) |
| Alternative/Functional Medicine Specialist | 7 (4.0%) | — | 7 (4.8%) |
| Alternative/Functional Medicine Specialist (ND) | 1 (0.6%) | — | 1 (0.7%) |
| Chiropractor (DC) | 1 (0.6%) | — | 1 (0.7%) |
| Unverified "Doctor" | 5 (2.9%) | — | 5 (3.4%) |
| Unverified "Doctor" (MD) | 1 (0.6%) | — | 1 (0.7%) |
| ^†^Values are n (%); ^‡^Other includes: (1) “High School Science Teacher,” (1) “Graduate Student” | | | |

**Supplementary Table 2.** Association between creator profession and health claim quality without unverified “doctors” (N = 158)

|  | Profession, n (%) | | PR (95% CI) | |
| --- | --- | --- | --- | --- |
| Health claim quality | Medical Creators,  N = 27 | Non-Medical Creators, N = 131 | Adjusted^a^ | Excluding Age^b^ |
| High | 16 (59.3%) | 5 (3.8%) | *Reference* | *Reference* |
| Poor or Moderate | 11 (40.7%) | 126 (96.2%) | **2.33 (1.5–3.62)** | **2.35 (1.51–3.65)** |
| Bold denotes statistical significance. PR: prevalence ratio; CI: confidence interval; a. adjusted for age, gender, year, quarter, and search query; b. adjusted for gender, year, quarter, and search query | | | | |

**Supplementary Table 3.** Association between creator profession and health claim quality without unverified “doctors” (N = 158)

|  | Profession, n (%) | | PR (95% CI) | |
| --- | --- | --- | --- | --- |
| Health claim quality | Medical Creators,  N = 27 | Non-Medical Creators, N = 131 | Crude | Adjusted^a^ |
| High | 16 (59.3%) | 5 (3.8%) | *Reference* | *Reference* |
| Poor or Moderate | 11 (40.7%) | 126 (96.2%) | **2.36 (1.5–3.73)** | **2.33 (1.5–3.62)** |
| Bold denotes statistical significance. PR: prevalence ratio; CI: confidence interval; a. adjusted for age, gender, year, quarter, and search query | | | | |

**Supplementary Table 4.** Associations between creator profession and monetization features without unverified “doctors” (N = 168)

|  | Profession, n (%) | | PR (95% CI)^a^ | |
| --- | --- | --- | --- | --- |
| Monetization feature | Medical Creators,  N = 29 | Non-Medical Creators, N = 139 | Crude | Adjusted^a^ |
| Any | 25 (86.2%) | 133 (95.7%) | 1.11 (0.96–1.29) | 1.13 (0.98–1.3) |
| Showcase | 12 (41.4%) | 91 (65.5%) | **1.58 (1.01–2.48)** | **1.66 (1.07–2.56)** |
| Subscription | 9 (31%) | 48 (34.5%) | 1.11 (0.62–2.01) | 1.18 (0.68–2.04) |
| External link | 21 (72.4%) | 101 (72.7%) | 1.01 (0.79–1.29) | 1.02 (0.79–1.32) |
| Bold denotes statistical significance. PR: prevalence ratio; CI: confidence interval; a. adjusted for age, gender, year, quarter, and search query | | | | |

**Supplementary Table 5.** Association between creator profession and health claim quality with unverified “doctors” categorized as physicians (N = 164)

|  | Profession, n (%) | | PR (95% CI) | |
| --- | --- | --- | --- | --- |
| Health claim quality | Medical Creators,  N = 33 | Non-Medical Creators, N = 131 | Crude | Adjusted^a^ |
| High | 16 (48.5%) | 5 (3.8%) | *Reference* | *Reference* |
| Poor or Moderate | 17 (51.5%) | 126 (96.2%) | **1.87 (1.34–2.6)** | **1.88 (1.37–2.58)** |
| Bold denotes statistical significance. PR: prevalence ratio; CI: confidence interval; a. adjusted for age, gender, year, quarter, and search query | | | | |

**Supplementary Table 6.** Associations between creator profession and monetization features with unverified “doctors” categorized as physicians (N = 174)

|  | Profession, n (%) | | PR (95% CI)^a^ | |
| --- | --- | --- | --- | --- |
| Monetization feature | Medical Creators,  N = 35 | Non-Medical Creators, N = 139 | Crude | Adjusted^a^ |
| Any | 25 (86.2%) | 135 (93.1%) | **1.24 (1.03–1.49)** | **1.24 (1.05–1.48)** |
| Showcase | 12 (41.4%) | 91 (62.8%) | **1.91 (1.19–3.07)** | **1.99 (1.25–3.15)** |
| Subscription | 9 (31%) | 50 (34.5%) | 1.1 (0.64–1.89) | 1.26 (0.74–2.12) |
| External link | 21 (72.4%) | 101 (70.1%) | 1.22 (0.91–1.63) | 1.17 (0.88–1.55) |
| Bold denotes statistical significance. PR: prevalence ratio; CI: confidence interval; a. adjusted for age, gender, year, quarter, and search query | | | | |

**Supplementary Table 7.** Sensitivity analysis of the association between creator profession and three-category health claim quality (N = 164)

|  | Profession, n (%) | | PR (95% CI) |
| --- | --- | --- | --- |
| Health claim quality | Medical Creators,  N = 33 | Non-Medical Creators, N = 131 | Adjusted^a^ |
| High | 16 (59.3%) | 5 (3.6%) | *Reference* |
| Moderate | 6 (22.2%) | 45 (32.8%) | **3.23 (1.58–6.62)** |
| Poor | 5 (18.5%) | 87 (63.5%) | **3.59 (1.69–7.61)** |
| Bold denotes statistical significance. PR: prevalence ratio; CI: confidence interval; a. adjusted for age, gender, year, quarter, and search query | | | |

**Supplementary Table 8.** Association between health claim quality and video likes adjusted for video-age (N = 164)

|  | *Ratio of expected likes, IRR (95% CI)* | | |
| --- | --- | --- | --- |
| Predictor | Crude | Adjusted^a^ | Video-age adjusted^b^ |
| Claim quality^†^ |  |  |  |
| High (n = 21) | *Reference* | *Reference* | *Reference* |
| Moderate (n = 51) | **3.04 (1.27–6.63)** | **3.16 (1.30–7.26)** | **3.59 (1.63–7.93)** |
| Poor (n = 92) | **3.8 (1.66–7.72)** | **3.37 (1.41–7.51)** | **4.20 (1.98–8.91)** |
| Bold denotes statistical significance. IRR: incidence rate ratio; CI: confidence interval; ^†^10 excluded due to not having health claims present in indexed video; a. adjusted for age, gender, year, quarter, search query, number of account followers, video subtitles, and whether or not the account had a Showcase; b. adjusted for age, gender, search query, number of account followers, video subtitles, log-transformed video age in days + 1, and whether or not the account had a Showcase | | | |
